# Effect of Telephone-Delivered Collaborative Goal Setting and Behavioral Activation vs Enhanced Usual Care for Depression Among Adults With Uncontrolled Diabetes: A Randomized Clinical Trial

**DOI:** 10.1001/jamanetworkopen.2019.8634

**Published:** 2019-08-07

**Authors:** Aanand D. Naik, Natalie E. Hundt, Elizabeth M. Vaughan, Nancy J. Petersen, Darrell Zeno, Mark E. Kunik, Jeffrey A. Cully

**Affiliations:** 1 Research Service Line, Houston Center for Innovations in Quality, Effectiveness, and Safety, Michael E. DeBakey VA Medical Center, Houston, Texas; 2 VA South Central Mental Illness Research, Education and Clinical Center, Michael E. DeBakey VA Medical Center, Houston, Texas; 3 Menninger Department of Psychiatry and Behavioral Sciences, Baylor College of Medicine, Houston, Texas; 4 Alkek Department of Medicine, Baylor College of Medicine, Houston, Texas

## Abstract

**Importance:**

Depression symptoms are present in one-third of patients with diabetes, contributing to significant adverse consequences. Population screening of high-risk patients coupled with telephone delivery of evidence-based therapies for comorbid diabetes may address barriers to care.

**Objective:**

To evaluate the effectiveness of proactive population screening plus telephone delivery of a collaborative goal-setting intervention among high-risk patients with uncontrolled diabetes and depression.

**Design, Setting, and Participants:**

In this randomized clinical trial, 225 participants (intervention [n = 136] and control [n = 89]) were enrolled from a regional Veterans Healthcare System serving Southeast Texas from November 1, 2012, through June 24, 2016. Data were gathered at baseline and 6 and 12 months after intervention. Patients selected had uncontrolled diabetes (hemoglobin A_1c_ [HbA_1c_] >7.5%]) and clinically significant depression (Patient Health Questionnaire–9 scores [PHQ-9] ≥10) and were living more than 20 miles from the Veterans Affairs medical center. Data collection was completed on December 6, 2016, and final analyses were completed by January 25, 2018. All analyses were intent to treat.

**Interventions:**

Healthy Outcomes Through Patient Empowerment (HOPE) included 9 telephone sessions with 24 trained health care professionals using collaborative goal-setting and behavioral activation methods. The control group received enhanced usual care (EUC) and notification of high-risk status.

**Main Outcomes and Measures:**

Change in depression symptoms using PHQ-9 and glycemic control using HbA_1c_ from baseline to 6 months and to 12 months. Secondary analyses evaluated clinically significant responses for these measures.

**Results:**

Among 225 participants, 202 (89.8%) were men, the mean (SD) age was 61.9 (8.3) years, 145 (64.4%) were married, and 156 (69.3%) had some education beyond high school. For the overall study, 38 participants (16.9%) were lost to follow-up or withdrew at 6 months and another 21 (9.3%) were lost to follow-up or withdrew at 12 months. Repeated-measures analysis with multiple imputation for missing data assessing the interaction of treatment group (HOPE vs EUC) and time (baseline, 6 months, and 12 months) found no significant improvement in PHQ-9 (β, 1.56; 95% CI, –0.68 to 3.81; *P* = .17) or HbA_1c_ (β, –0.005; 95% CI, –0.73 to 0.72; *P* = .82). Analyses using *t* test for change from baseline to 12 months showed a HOPE vs EUC between-group mean difference for PHQ-9 of 2.14 (95% CI, 0.18 to 4.10; *P* = .03) and for HbA_1c_ of –0.06% (95% CI, –0.61% to 0.50%; *P* = .83). A secondary analysis of patients experiencing a clinical response found that 52.1% of HOPE participants had clinically significant responses in PHQ-9 at 12 months vs 32.9% in EUC (difference, 0.19; 95% CI, 0.04-0.33; *P* = .01).

**Conclusions and Relevance:**

Telephone-delivered, collaborative goal setting produced clinically significant reductions in depression symptoms but not glycemic control among patients who remained engaged at 12 months compared with EUC among a population screened sample of high-risk patients with diabetes and depression. Although the intervention created some lasting effect for depression, additional strategies are needed to maintain engagement of this high-risk population within an interprofessional team approach to primary care.

**Trial Registration:**

ClinicalTrials.gov identifier: NCT01572389

## Key Points

**Question:**

Can a telephone-delivered, collaborative goal-setting intervention improve depression symptoms and glycemic control among high-risk, patients with comorbid diabetes identified using a population screening approach?

**Findings:**

In this randomized clinical trial of 225 US veterans with uncontrolled diabetes and significant depression symptoms, a telephone-delivered intervention using collaborative goal setting and behavioral activation had mixed results compared with enhanced usual care. Secondary analyses found that a significantly higher proportion of intervention participants achieved and maintained clinically significant responses of depression symptoms at 12 months but did not find such improvements for glycemic levels at 12 months.

**Meaning:**

A telephone-delivered, collaborative goal-setting approach can improve depression symptoms among high-risk patients with uncontrolled diabetes who maintain engagement for 12 months.

## Teaser

This randomized clinical trial compares the effectiveness of telephone-delivered collaborative goal setting and behavioral activation vs enhanced usual care for depression symptoms among US veterans with uncontrolled diabetes.

## Introduction

Approximately one-third of patients with diabetes have clinically significant depression symptoms, contributing to substantial adverse consequences.^1^ Patients with diabetes and depression use more health services and have greater functional impairment and morbidity compared with those without depression.^2,3,4^ Depression increases mortality risks among patients with diabetes, especially older adults and those who have had recent cardiovascular events.^5^ Furthermore, there are correlations between the number of diabetes-related symptoms and the number of depression symptoms, suggesting a magnification of physical and affective symptoms in patients with comorbidities.^6^

Evidence-based psychotherapies, pharmacologic therapy, and coordinated or stepped-care approaches have moderate efficacy in reducing depression symptoms and modest success with improving hemoglobin A_1c_ (HbA_1c_) and diabetes self-care activities.^5,7,8^ However, few studies^7,9,10^ have addressed the needs of complex medically ill populations regarding sustained treatment and follow-up periods.^7,9,10^ Evidence-based treatments for comorbid diabetes and depression are limited because of scarcity of skilled health professionals, need for frequent visits, and difficulty identifying who could benefit from mental health treatment.^9,11^ A previous study^6^ reported that individuals living more than 10 miles from their health care facility had significantly fewer follow-up visits for mental health. Furthermore, identification of at-risk patients is often difficult using customary screening methods.^11,12^ Learning health care systems using electronic health records linked to large data repositories provide digital infrastructure for screening high-risk populations using more efficient and reliable methods.^13^ Learning health care systems can use structured clinical, billing, and outcomes data to identify clusters of patients with persistently uncontrolled diabetes and depression risk factors before they present with more serious illness.^14^

Systems approaches^15^ to identifying high-risk populations with depression and diabetes are essential^11,16^ but should be coupled with pathways to evidence-based treatments.^5^ Structured telephone delivery may address some barriers to accessing effective treatments for diabetes self-care and depression symptoms, especially for rural-living and functionally impaired individuals.^17^ Three meta-analyses reported that telehealth delivery (call or text message) provides small but significant improvements in glycemic control among patients with diabetes.^18,19,20^ Furthermore, a few studies^10,21,22^ incorporating telehealth approaches showed improvements in depression and anxiety symptoms among patients with diabetes. However, additional research is needed to evaluate whether structured, telephone-delivered behavioral interventions can enhance treatment for depression symptoms and glycemic control for high-risk patients with comorbidity.^10^

A previous study^23^ showed the effectiveness of collaborative goal setting as a behavioral intervention for glycemic control in chronically ill patients. Collaborative goal setting has 4 key components: (1) identifying what matters most to patients (health-related values); (2) using these values to set specific, measurable, and actionable health outcome goals; (3) communicating outcome goals to the patient’s clinicians; and (4) working with clinicians to align reasonable treatment and self-care recommendations to achieve outcome goals. Collaborative goal setting is an effective, guidelines-concordant strategy in diabetes care.^24,25,26,27^ Goal setting promotes behavioral activation, an evidence-based psychotherapy,^28^ which acts synergistically to reduce depression symptoms and enhance self-care.^29,30,31^

To address barriers to screening, access, and treatment of high-risk patients with diabetes and depression, we developed the Healthy Outcomes Through Patient Empowerment (HOPE) intervention.^31^ This study used a learning health care systems approach^14,15^ to screen for and improve treatment of a high-risk subpopulation. The HOPE approach provides a structured, telephone-delivered, collaborative goal-setting intervention for 6 months to enhance behavioral activation targeting depression symptoms and diabetes self-care. The objective of this study was to evaluate the effectiveness of HOPE for clinically significant improvements in depression and glycemic control compared with enhanced usual care (EUC)—usual diabetes and depression care enhanced by a systems approach to screening for high-risk status.^13,32,33^

## Methods

### Study Design

This randomized clinical trial, conducted from November 1, 2012, through June 24, 2016, examined a telehealth intervention for patients with uncontrolled diabetes and clinically significant depression.^32^ The trial protocol can be found in Supplement 1. The Baylor College of Medicine institutional review board and the Michael E. DeBakey VA Medical Center (MEDVAMC) research and development committee, both in Houston, Texas, approved the study protocol. All participants provided verbal informed consent conducted by telephone. The study was reported in accordance with the Consolidated Standards of Reporting Trials (CONSORT) reporting guideline. Data collection was completed on December 6, 2016, and final analyses were completed by January 25, 2018. All analyses were intent to treat.

The trial used an integrated, regional Veterans Affairs (VA) learning health care system.^13,14,33^ This approach consists of (1) an electronic data warehouse to identify a specific high-risk population (veterans with persistent uncontrolled diabetes and depression risk factors living at least 20 miles from the medical center), followed by (2) telephone screening for depression using the Patient Health Questionnaire–9 (PHQ-9) and (3) training of clinicians to deliver a structured telehealth intervention that can be counted as a routine clinical encounter. Enrolled participants were randomized to a 6-month blended diabetes and depression behavioral health coaching program, followed by a 6-month maintenance period without coaching (intervention), or received usual clinical care plus educational materials (EUC) for 12 months. Primary outcomes were glycemic control, as measured by HbA_1c_, and depression control, as measured by the PHQ-9.^34,35^

### Participants and Eligibility Criteria

**Figure 1.**
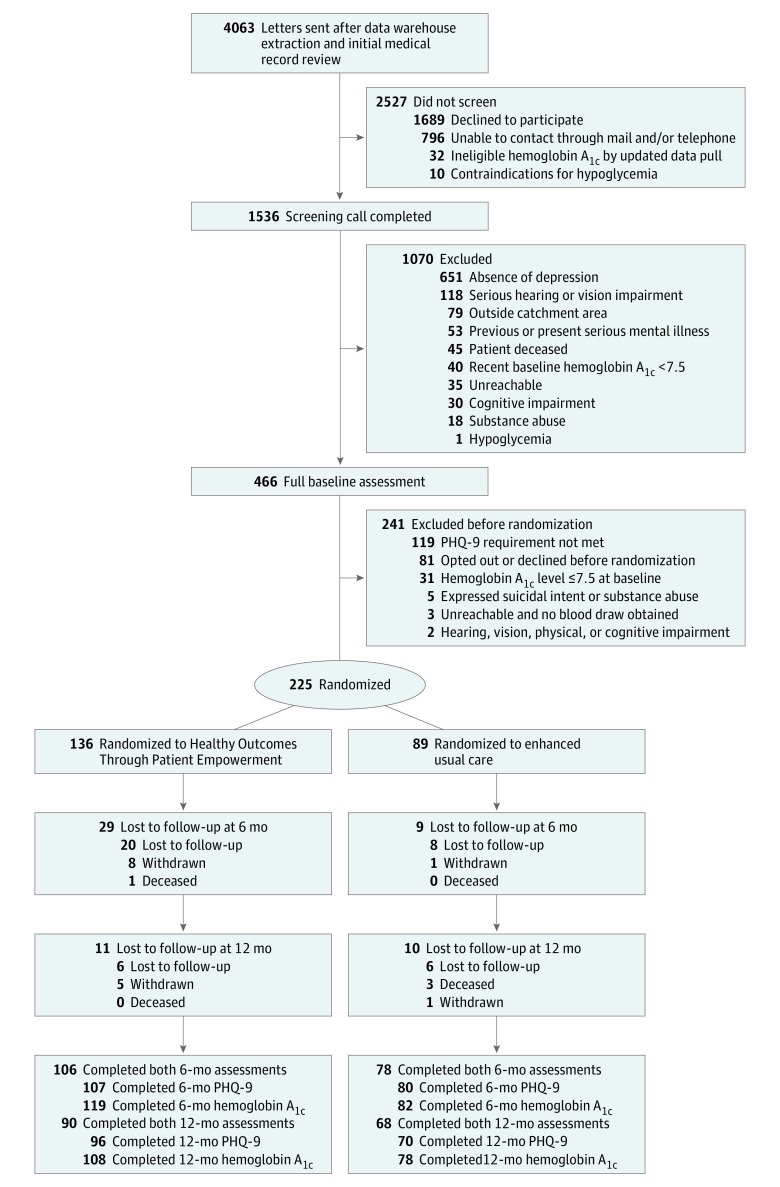
CONSORT Diagram of Patient Flow PHQ-9 indicates Patient Health Questionnaire–9.

The study was conducted at the MEDVAMC and 6 affiliated community-based outpatient clinics across Southeast Texas. A corporate data warehouse was used to identity 4063 veterans with uncontrolled diabetes (defined by *International Classification of Diseases, Ninth Revision* diagnosis code 250.XX and HbA_1c_ of ≥7.5% for 1 year before the study) who lived at least 20 miles from the main Veterans Health Administration hospital in Houston, Texas, or who received primary care services within a MEDVAMC satellite community-based clinic across Southeast Texas (Figure 1). Among patients expressing interest, medical record and telephone screening (n = 1536) was conducted. Patients were excluded during this screening if there was an absence of depression symptoms, a telephone-based coaching intervention would be inappropriate (eg, the patient had severe cognitive impairment or mental health condition, hearing or visual impairment, or active suicidal ideation), or presence of significant hypoglycemic events or substance abuse. Among those not initially excluded, 466 completed a full baseline assessment by telephone to confirm inclusion criteria eligibility for clinically significant depression symptoms (score ≥10 on the PHQ-9)^4,5^ and uncontrolled diabetes at baseline (HbA_1c_ >7.5%).^36,37,38^ Of these, 225 patients met all eligibility criteria and consented to randomization (Figure 1).

### Randomization and Blinding

To increase statistical power for measuring the intervention and factors related to its telehealth delivery in secondary treatment analyses, the final sample was assigned to treatment groups using unequal block randomization (60% intervention [n = 136] and 40% control [n = 89]), stratified by clinic site. Independent evaluators, blinded to group assignment, gathered baseline, 6-month, and 12-month data by telephone and were not involved in any other study procedures. At each respective time point, evaluators scheduled participants for blood sample collection and HbA_1c_ measurement using standardized methods at a local VA clinical laboratory. If participants could not participate at 1 or more collection point, HbA_1c_ data were extracted from medical records if values were present within 4 weeks of the expected time frame. Research staff enrolled and assigned patients to groups using a blinded randomization procedure.

### Interventions

#### HOPE Participants

During the active intervention, the HOPE group received 9 coaching sessions with a trained health professional: biweekly (for 30-40 minutes) from months 1 to 3 and monthly (for 15 minutes) from months 4 to 6. Twenty-four trained health professionals or coaches (18 female) included psychologists (n = 16), nurses (n = 5), pharmacists (n = 2), and social workers (n = 1). Most (n = 18) were at the MEDVAMC; 6 were at a Veterans Health Administration community-based clinic. Coaches were assigned primarily by similar site of care and availability. Clinician training consisted of facilitated training (two 120-minute teleconference sessions) and subsequent support (mentoring and feedback related to fidelity to the intervention) from a behavioral health expert (N.E.H.), plus monthly peer mentoring with other coaches. Patients and coaches used workbooks that guided telephone conversations and allowed patients to define and track their progress. Primary care physicians received notifications of their patients’ participation, HbA_1c_ results, and PHQ-9 questionnaire outcomes via secure electronic messaging; however, they received no formal training related to the HOPE intervention components.

During the first 2 patient sessions, HOPE coaches focused on building rapport, introducing and clarifying values, collaboratively setting initial goals, identifying potential skill sets to address goals, and empowering patients to advocate for their health through active communication with their clinicians. For sessions 3 through 6, participants focused on discrete skill modules (increasing pleasant activities, using thoughts to improve wellness, diet, physical activity, medication management, and relaxation)^3^ customized to meet their diabetes and depression goals. Sessions 7 through 9 focused on maintenance skills (reviewing action plans and overcoming barriers).^32^ Skills emphasized in the modules were designed to improve diabetes- and depression-related outcomes simultaneously. The HOPE modules stressed the importance of the coach-patient relationship as critical to improvement in participant physical and/or emotional self-management. During months 7 to 12, participants received usual primary care without contact from HOPE coaches.

#### Enhanced Usual Care

In addition to usual care, EUC participants were informed about their high-risk status (uncontrolled diabetes status and clinically significant depression symptoms) and were given related educational materials. Study assessments were conducted for EUC participants via telephone, and educational materials were mailed. Participants were encouraged to address these results with their primary care clinician.

#### Telehealth

Investigators defined telehealth in concordance with the American Telemedicine Association guideline for delivering clinical services by using remote technology (eg, telephone).^39^ Because of the difficulty for participants to commute to study sites, all procedures (screening, enrollment, protocol delivery, and follow-up assessments) were conducted by telephone to ease treatment burden. Participants from both study arms visited the study site only to obtain HbA_1c_ measurements and their usual medical care.

### Outcomes

Primary outcomes included change in glycemic control (HbA_1c_) and depression symptoms (PHQ-9) from baseline to 6 months and 12 months. As secondary outcomes, patients with HbA_1c_ response were those with a 0.5% decrease in HbA_1c_ (as the minimal clinically significant improvement) from baseline.^40^ Those with PHQ-9 response included those with at least 50% decrease from baseline or a PHQ-9 value less than 10.^36,37,38^ In addition, other variables, including demographics, health care use, self-efficacy, comorbid physical and mental conditions, and other psychological factors, were evaluated.

### Power Analysis

To achieve 80% power to detect an effect size of 0.45 at α = .05, 242 participants were needed. Unequal randomization (60 intervention to 40 control) was used, and attrition at 12 months was assumed to be 25%.

### Statistical Analysis

Analyses were performed based on the randomized treatment assignment and not on the treatment as received. Descriptive statistics were used to characterize differences by study arm at baseline. Means (SDs) were tabulated separately for EUC and HOPE for HbA_1c_ and PHQ-9 scores at baseline, 6 months, and 12 months. Contrasts between baseline, 6 months, and 12 months were tested for differences in outcomes at the follow-up times. A 2-tailed *P* < .05 was considered to be statistically significant.

To test for differences in the means over time between groups for each outcome, we used a pooled *t* test for equal variances or Satterthwaite approximation for unequal variances. A significant value indicated that the outcome changed significantly within the group during these periods. We also conducted a repeated measures analysis with participants nested within site, while accounting for all study time points within a single model for HbA_1c_ and a single model for PHQ-9 outcomes. Participants with missing values were included with multiple imputation procedures using Monte Carlo simulations to account for missing values. Separate models for HbA_1c_ and PHQ-9 outcomes account for each of the periods (baseline, 6 months, and 12 months). The independent variables in the model were time, treatment, and the interaction of time and treatment. Significant time-by-treatment interactions indicate a significant difference between the HOPE and EUC groups during the 12 months.

In our secondary analyses, change scores for the outcomes were derived and tested for differences between HOPE and EUC. The 6-month change score for each patient was calculated as the 6-month value minus the baseline value. A negative value indicates that the patient had less depression at 6 months compared with baseline. The success rate difference (SRD) was calculated as *p_1_ – p_2_*, where *p_1_* was the proportion of responders from HOPE and *p_2_* was the proportion of responders in EUC. An SRD more than 0 indicated that HOPE was preferred to EUC.^41^ Based on previous studies,^41,42,43^ treatment responses with CIs were categorized as small (SRD, 0.11; number needed to treat [NNT] = 9), medium (SRD, 0.28; NNT = 4), and large (SRD, 0.43; NNT = 2).

## Results

Of 225 participants randomized, 136 were assigned to HOPE and 89 were assigned to EUC. For the overall study, 38 (16.9%) participants were lost or withdrew at 6 months and another 21 (9.3%) were lost or withdrew at 12 months. Figure 1 describes differences by study arm. A higher percentage of EUC participants completed assessments at both 6 and 12 months. Participants who completed the study at 12 months reported higher diabetes knowledge, diabetes self-care understanding, and health literacy. There were no differences in sociodemographic or other clinical variables at baseline among participants who completed and did not complete the study at 12 months.

### Characteristics of the Sample

Table 1 shows demographic characteristics of participants at baseline. Of 225 participants, most were older (mean age [SD], 61.9 [8.3] years), male (202 [89.8%]), married (145 [64.4%]), educated beyond high school (155 [68.9%]), and retired (74 [32.9%]) or disabled (92 [40.9%]). Most participants rated their health as fair to poor (173 [76.9%]) and had poorly controlled diabetes (mean [SD] HbA_1c_, 9.3% [1.4%]) and moderately severe depression symptoms (mean [SD] PHQ-9, 15.9 [4.1]). Groups did not significantly differ at baseline in terms of age, race/ethnicity, occupation, HbA_1c_ levels, PHQ-9 severity and categories, Deyo comorbidity scores,^44^ and insulin use.

**Table 1.** Demographic Characteristics of Participants 6 Months Before Baseline Measures

Characteristic	No. (%)		
	Total (N = 225)	HOPE (n = 136)	EUC (n = 89)
Age ≥65 y	104 (46.2)	68 (50.0)	36 (40.4)
Male	202 (89.8)	121 (89.0)	81 (91.0)
Marital status			
Single	17 (7.6)	10 (7.4)	7 (7.9)
Married	145 (64.4)	87 (64.0)	58 (65.2)
Separated or divorced	63 (28.0)	39 (28.7)	24 (27.0)
Race/ethnicity			
White	124 (55.1)	73 (53.7)	51 (57.3)
Non-Hispanic black	57 (25.3)	41 (30.1)	16 (18.0)
Hispanic	23 (10.2)	12 (8.8)	11 (12.4)
Other	21 (9.3)	10 (7.4)	11 (12.4)
Lives alone at home	41 (18.2)	26 (19.1)	15 (16.9)
Education			
High school or less	69 (30.7)	38 (27.9)	31 (34.8)
Some college or college graduate	148 (65.8)	92 (67.6)	56 (62.9)
Graduate school	7 (3.1)	5 (3.7)	2 (2.2)
Household income, $			
<30 000	109 (48.4)	59 (43.4)	50 (56.2)
30 000-59 999	89 (39.6)	61 (44.9)	28 (31.5)
≥60 000	24 (10.7)	15 (11.0)	9 (10.1)
Employment			
Employed full- or part-time	38 (16.9)	22 (16.2)	16 (18.0)
Retired	74 (32.9)	48 (35.3)	26 (29.2)
Disabled	92 (40.9)	56 (41.2)	36 (40.4)
Other	20 (8.9)	9 (6.6)	11 (12.4)
Patient-rated health			
Excellent to very good	5 (2.2)	3 (2.2)	2 (2.2)
Good	45 (20.0)	30 (22.1)	15 (16.9)
Fair to poor	173 (76.9)	101 (74.3)	72 (80.9)
Diabetes management			
Insulin only	60 (26.7)	36 (26.5)	24 (27.0)
Oral agents	61 (27.1)	38 (27.9)	23 (25.8)
Insulin and oral agents	62 (27.6)	32 (23.5)	30 (33.7)
Lifestyle only	42 (18.7)	30 (22.1)	12 (13.5)
PHQ-9 scores			
10-14	95 (42.2)	61 (44.9)	34 (38.2)
15-19	80 (35.6)	42 (30.9)	38 (42.7)
>19	50 (22.2)	33 (24.3)	17 (19.1)
PHQ-9 severity scores, mean (SD)	15.9 (4.1)	15.8 (4.2)	16.2 (4.0)
HbA_1c_ level, mean (SD)	9.3 (1.4)	9.2 (1.4)	9.3 (1.5)
Deyo comorbidity score, mean (SD)	2.1 (1.6)	2.1 (1.5)	2.1 (1.8)

Abbreviations: EUC, enhanced usual care; HbA_1c_, hemoglobin A_1c_; HOPE, Healthy Outcomes Through Patient Empowerment; PHQ-9, 9-item Patient Heath Questionnaire.

### Primary Outcomes

**Figure 2.**
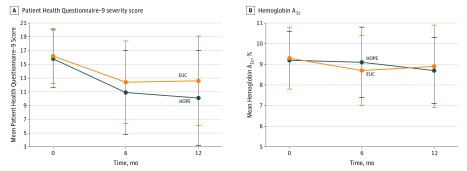
Mean Quantitative Values for Patient Health Questionnaire–9 and Hemoglobin A_1c_ From Baseline to 12-Month Follow-up HOPE indicates Healthy Outcomes Through Patient Empowerment; EUC, enhanced usual care; and error bars, SD.

Figure 2 shows the change in primary outcomes for PHQ-9 and HbA_1c_ across 3 time points stratified by HOPE and EUC groups. The HOPE participants experienced a decline in depression symptoms (PHQ-9) from baseline (mean [SD], 15.8 [4.2]) to 6 months (10.9 [6.1]), which persisted at 12 months (10.1 [6.9]). The EUC participants had a similar but more modest change from baseline (16.2 [4.0]) to 6 months (12.4 [6.0]) and 12 months (12.6 [6.5]). The PHQ-9 differences between HOPE and EUC were statistically significant at 6 months (mean difference, 1.74; 95% CI, 0.14-3.33; *P* = .03) and 12 months (mean difference, 2.14; 95% CI, 0.18-4.10; *P* = .03). Figure 2B shows that HOPE participants had improved glycemic control (mean [SD] HbA_1c_) from baseline (9.2% [1.4%]) to 6 months (9.1% [1.7%]) and 12 months (8.7% [1.6%]). The EUC participants experienced a decline in mean (SD) HbA_1c_ from baseline (9.3% [1.5%]) to 6 months (8.7% [1.7%]) and then a gradual regression at 12 months (8.9% [2.0%]). Differences in HbA_1c_ between groups were not significant at 6 months (mean difference, –0.40%; 95% CI, –0.86% to 0.06%; *P* = .08) or 12 months (mean difference, –0.06%; 95% CI, –0.61% to 0.50%; *P* = .83). Half (51%) of the HOPE participants attended 6 or more sessions, and 73% attended 3 or more sessions. Session attendance did not have a significant effect on the primary outcomes. Repeated-measures analysis with multiple imputation for missing data assessing the interaction of treatment group and time revealed no significant improvement in PHQ-9 (β, 1.56; 95% CI, –0.68 to 3.81; *P* = .17) or HbA_1c_ (β, –0.005; 95% CI, –0.73 to 0.72; *P* = .82).

### Secondary Outcomes

Table 2 shows the number of participants experiencing a clinical response in HbA_1c_ and PHQ-9 at 6 months and 12 months and compares success rates between groups. At 6 months, 38% (47.2% in the HOPE group and 35.0% in the EUC group) of all participants had improved PHQ-9 in both groups. Although not statistically significant, this was a small effect size (success rate difference, 0.12; 95% CI, –0.02 to 0.26; *P* = .09) for HOPE at 6 months, with an NNT of 8. At 12 months, there were significantly more patients with PHQ-9 response in the HOPE group (52.1%) than in the EUC group (32.9%) (success rate difference, 0.19; 95% CI, 0.04 to 0.33; *P* = .01) with an NNT of 5. At 6 months, there were more patients with an HbA_1c_ response in the EUC group (57.7%) than in the HOPE group (37.7%; success rate difference, –0.20; 95% CI, 0.05 to 0.33; *P* = .007). By 12 months, there were no significant differences in HbA_1c_ response between the HOPE group (48.9%) and EUC group (51.5%; success rate difference, –0.03; 95% CI, –0.13 to 0.18; *P* = .75); however, half (50.0%) of all participants, regardless of group, had clinically significant improved glycemic control.

**Table 2.** Comparison of Participants With HbA_1c_ and PHQ-9 Response at 6 and 12 Months in the HOPE Intervention and EUC Groups^a^

Measure	No./Total No. (%)		*P* Value	Success Rate Difference (95% CI)^b^	No. Needed to Treat
	HOPE	EUC			
**6-mo Response**					
HbA_1c_	40/106 (37.7)	45/78 (57.7)	.01	−0.199 (0.05 to 0.33)	5
PHQ-9	51/108 (47.2)	28/80 (35.0)	.09	0.12 (−0.02 to 0.26)	8
**12-mo Response**					
HbA_1c_	44/90 (48.9)	35/68 (51.5)	.75	−0.026 (−0.13 to 0.18)	38
PHQ-9	50/96 (52.1)	23/70 (32.9)	.01	0.19 (0.04 to 0.33)	5

Abbreviations: EUC, enhanced usual care; HbA_1c_, hemoglobin A_1c_; HOPE, Healthy Outcomes Through Patient Empowerment; PHQ-9, 9-item Patient Health Questionnaire.

^a^ A participant with HbA_1c_ response was defined as an individual with a decrease of 0.5 in HbA_1C_ from baseline; PHQ-9 response was defined as a change of at least 50% from baseline or a value of PHQ-9 less than 10.

^b^ Success rate difference was calculated as *p_1_ – p_2_*, where *p_1_* is the proportion of HOPE participants with response and *p_2_* is the proportion of EUC group participants with response. A success rate difference greater than 0 indicates that, overall, the HOPE treatment was preferred to the EUC condition.

Table 3 shows health care use among participants in the HOPE group compared with the EUC group. At baseline, there was a significant difference between the HOPE and EUC groups for the percentage of patients who were prescribed mental health medications (χ^2^ = 4.65; *P* = .03) but no difference for mental health clinic visits, primary care visits, or diabetes medication prescriptions. There was substantially less active medication management (increase, decrease, or stop medications) for mental health compared with diabetes medications for both groups. There were no statistically significant differences in active management between groups. In both, the number of mental health and primary care visits was comparable throughout the study.

**Table 3.** Health Care Use Among HOPE and EUC Participants at Baseline, 6 Months, and 12 Months

Health Care Service	No. (%)					
	Baseline (n = 225)^a^		6 mo (n = 188)^b^		12 mo (n = 166)^c^	
	HOPE (n = 136)	EUC (n = 89)	HOPE (n = 108)	EUC (n = 80)	HOPE (n = 96)	EUC (n = 70)
**Mental Health Management**						
Patients prescribed mental health medications^d^	49 (36.0)	20 (22.4)	NA	NA	NA	NA
Mental health active management^e^	NA	NA	14 (13.0)	7 (8.8)	5 (5.2)	9 (12.9)
Mental health clinic visits	61 (44.9)	38 (42.7)	53 (49.1)	34 (42.5)	45 (46.9)	35 (50.0)
**Diabetes Management**						
Patients prescribed diabetes medications	106 (77.9)	77 (86.5)	NA	NA	NA	NA
Diabetes active management^e^	NA	NA	43 (40.0)	37 (46.2)	26 (27.1)	23 (32.9)
Primary care clinic visits	119 (87.5)	76 (85.4)	97 (90.0)	73 (91.2)	89 (92.7)	64 (91.4)

Abbreviations: EUC, enhanced usual care; HOPE, Healthy Outcomes Through Patient Empowerment; NA, not applicable.

^a^ Baseline data represented the period of 181 days before baseline through baseline.

^b^ Six-month data represented the period of day 1 through day 181.

^c^ Twelve-month data represented the period of day 182 through day 365.

^d^ At baseline, there was a significant difference (*P* = .03) between HOPE and EUC groups.

^e^ Active management was defined as any change in medication compared with baseline (ie, switch from 1 medication to another, addition of a new medication, discontinuation of a medication, uptitration, or downtitration).

## Discussion

This randomized clinical effectiveness trial showed that a collaborative goal-setting intervention delivered by trained health professionals (HOPE) provided mixed results, with indications of an effect on depression symptoms and little to no effect on glycemic control in a sample of high-risk patients with comorbid uncontrolled diabetes compared with usual care enhanced by a systems approach to screening for high-risk status. Among participants who completed the study at 12 months, we found clinically and statistically significant improvements in depression symptoms compared with EUC. Fifty-two percent of HOPE participants responded to treatment of depression symptoms. Participants in both study arms experienced modest improvements in glycemic control at 12 months, with no difference between groups.

Recent meta-analyses found that psychotherapy was effective (standardized mean differences [SMD], –0.64 to –1.47) at reducing depression symptoms but had mixed efficacy (SMD, 0.4 to –1.40) for glycemic control.^5,45,46^ A systematic review of collaborative care (ie, case manager and structured treatment plans) for depression found similar results for depression symptoms (SMD, –0.13 to –0.68) and glycemic control (SMD, 0 to –0.54).^7^ Within this context, the findings of the current study were comparatively less effective but within the range of the existing literature. Although the findings of the multiple imputation analyses were disappointing, the improvements in clinically significant depression response were more positive and consistent with previous literature.^5,7,45,46^

Among previous studies,^10,47^ relatively few clinical trials used significant or exclusive telephone delivery of psychotherapy for depression with comorbid diabetes. Piette et al^10^ found that telephone-delivered psychotherapy for patients with comorbid diabetes was significantly more likely to promote depression remission (58%) at 12 months compared with usual care (39%) but did not improve glycemic control. Furthermore, a meta-analysis of 5 clinical trials found that telephone interventions for glycemic control were not more effective than usual diabetes care (mean HbA_1c_ difference, −0.91% to 0.16%).^18^ The results of the current study underscore the difficulties of reaching and delivering care to a population by telephone who live at some distance from specialty diabetes and mental health care services. Overall, the telephone-delivered intervention had mixed results, with data showing positive effects on depression with limited effects on glycemic control compared with enhanced usual care. Patients who remained engaged in the intervention at 12 months experienced significantly higher levels of depression response compared with usual care, which is consistent with the aforementioned study.^10^ Given the consistency in findings across studies, future efforts to address complex, multimorbid patient populations should explore options for more robust intervention efforts and delivery platforms. For example, the HOPE intervention was not closely aligned with ongoing clinical care activities outside the study protocol. Future efforts to systematically align psychosocial and behavioral intervention efforts with ongoing clinical care practices through interprofessional teams may improve immediate and longer-term outcomes for complex patient populations.^48,49^

As part of these efforts, a collaborative goal-setting approach building on the one used in the present trial may improve alignment between clinicians to address the multifaceted goals and priorities of multimorbid patients with complex conditions.^48,50,51,52^ In the current trial, EUC participants experienced regression toward the mean for both PHQ-9 scores and HbA_1c_, HOPE participants experienced sustained or even improved clinical responses at 12 months. These results reproduce findings from other studies^23,30^ suggesting that collaborative goal setting can produce lasting behavioral change among patients who remain engaged in the intervention.

The results seen in the present study may offer some support for learning health care system approaches.^13,14,33^ For the present study, this approach used precision algorithms to identify high-risk patients and then link those populations to robust diabetes and depression care within a patient-centered medical home model.^5^ Within this model, EUC participants achieved similar levels of glycemic control as HOPE participants, and 32.9% had a clinical response for their depression.

### Limitations

Participants were largely men and drawn from 1 regional area. This may limit the generalizability of our findings to other patient populations. Similarly, the Veterans Health Administration is a national, integrated health care system with a robust infrastructure for population health and mental health care that may not be available to non–Veterans Health Administration populations. However, further coordination among primary care clinicians and HOPE coaches might have produced better glycemic control. The intervention group had a greater participation burden compared with the EUC group, which may have contributed to higher attrition rates during the follow-up periods in comparison with studies that use attention controls with similar participation requirements across intervention and control groups. The study did not reach its recruitment target at baseline, which may have affected some analyses given dropout and missing data at 12 months.

## Conclusions

This study showed that HOPE, a telehealth intervention using collaborative goal setting and behavioral activation, may potentially improve depression symptoms for patients with diabetes and comorbid depression, especially for those who complete the intervention process. Its effects on glycemic control were negative. These results provide some support for population-based screening of high-risk patients with diabetes and comorbid depressive symptoms for telephone delivery of care for chronic conditions. Future studies should build on the approach and findings of HOPE in other low-income or rural settings where access to integrated diabetes and depression care is sparse.

## Notes

Supplement 1. **Trial Protocol**

Supplement 2. **Data Sharing Statement**
