# Supplementary material for: Effect of Telephone-Delivered Collaborative Goal Setting and Behavioral Activation vs Enhanced Usual Care for Depression Among Adults With Uncontrolled Diabetes: A Randomized Clinical Trial
Source: JAMA Netw Open. 2019 Aug 7;2(8):e198634. doi: 10.1001/jamanetworkopen.2019.8634 (PMC6686779; doi:10.1001/jamanetworkopen.2019.8634)
Supplement: Supplement 1. — Trial Protocol [file jamanetwopen-2-e198634-s001.pdf]

✓Compare

Institutional Review Board for Baylor College of Medicine and Affiliated Hospitals

Protocol Number: H-30139
Status: Approved
Initial Submit Date: 12/21/2011
Approval Period: 8/27/2018 - 8/26/2019

Section Aa: Title & PI

A1. Main Title

BEHAVIORAL ACTIVATION THERAPY FOR RURAL VETERANS WITH DIABETES AND DEPRESSION

A3a. Financial Conflict of Interest

Does any member of study personnel (Investigator (including investigator’s spouse and/or dependent children)) that are involved in the design, conduct, or reporting of the research have a Significant Financial Interest (SFI) that would reasonably appear to be affected by the research for which funding is sought and/or associated with an entity/business that would reasonably appear to be affected by the research?

No

Section Ab: General Information

A5. Funding Source:

Organization: VA HSR&D

A6a. Institution(s) where work will be performed:

BCM: Baylor College of Medicine
Michael E. DeBakey Veterans Affairs Medical Center

A6b. Research conducted outside of the United States:

Country:
Facility/Institution:
Contact/Investigator:
Phone Number:

If documentation of assurances has not been sent to the Office of Research, please explain:

A7. Research Category:

A8. Therapeutic Intent

Does this trial have therapeutic intent?
Yes

Section B: Exempt Request

B. Exempt From IRB Review

Not Applicable

Section C: Background Information

Co-occurrence of diabetes and clinically significant depressive symptoms is highly prevalent, with grave consequences for quality of life and health. Almost one third of diabetic patients have clinically significant symptoms of depression. They use health services more, experience greater functional impairment, and have higher morbidity and mortality than diabetic patients without depression. Furthermore, depression is associated with greater non-adherence to diabetes self-care. Due to the complex interrelation between diabetes and depression, patients need focused interventions that blend physical and emotional health strategies and interface through existing clinical-care settings, such as VA Patient Aligned Care Teams (PACT).

Provision of comprehensive care for diabetes and depression in primary care is limited due to system-, provider-, and patient-related barriers, exacerbated in rural settings due to limited availability of providers (especially mental health specialists), patient concerns about mental health treatment, and logistic issues, such as distance to VA services. Our prior work suggests that the interrelation of diabetes and depression provides an opportunity to treat both simultaneously, using individualized behavioral coaching to activate goal setting and positive behaviors (e.g., adhering to medication regimens, managing diet, and increasing pleasant events). Our intervention involves telephone interactions to increase access for Veterans residing at significant distances from a VA facility. In this proposal, behavioral activation describes intervention content, founded on behavior-change techniques using goal setting and action planning. Behavioral coaching describes the intervention, which uses non-expert providers (coaches) to implement treatment.

Approximately 3 million Veterans live in rural areas and have numerous health-related challenges, including lower health-related quality of life, more comorbidites, and limited access to health care services than their urban or suburban counterparts. Rural-dwelling veterans with diabetes and depression are typically treated in community-based outpatient clinics (CBOCs). Therefore, to be effective; treatment modalities for veterans must meet the demands of community-based care. This is especially true for patients who reside in rural settings where medical and psychological services are scarce and difficult to obtain. These conditions are not well managed in rural areas due to various barriers to care, including distance/ transportation, limited health care supply, quality of health care, financial constraints, and social isolation. Diabetic patients in rural areas tend to have more problems controlling their blood sugar, blood pressure and cholesterol compared to urban

patients, thereby increasing their risk of diabetic complications. Similarly, rural patients with depression have similar barriers to care that increase their risk of poorer health outcomes as well.

Current methods of treating diabetic patients with depression require the use of clinical services that are difficult to access outside of tertiary care centers. Access to certified diabetes educators, nutritionists, and mental health clinicians is limited in community-based and rural settings. Too little commitment has been given to the application of blended behavioral therapies that target the physical and emotional health needs of patients with diabetes and depression in CBOCs. Furthermore, these interventions may be more effective if they utilize methods that can better reach rural-dwelling Veterans such as home-based and telephone mediated therapeutic sessions. Training and utilizing behavioral health coaches (BHCs) to deliver telephone-mediated therapies may enhance the reach of treatments for co-occurring diabetes and depression. The implementation of such treatments will require the development and testing of therapeutic manuals and BHC training protocols to ensure standardization and effectiveness.

Section D: Purpose and Objectives

The overall objective of this study is to improve the effectiveness of care for Veterans with diabetes and depression receiving care in VA community-based outpatient clinics (CBOCs) or living at some distance from tertiary care. Given our experiences, we do not believe that effectiveness can be achieved simply through dissemination of research findings to PCPs or CBOCs using standard channels. The current proposal has obtained funding to integrate our prior work which developed methods for conducting a behavioral coaching intervention including training coaches to administer a standardized treatment protocol, and measuring the development of high-quality behavioral goals and action plans. Our prior work , which included pilot data for the proposed HOPE study, demonstrated that behavioral coaching interventions can improve clinical outcomes in patients such as: lowered HbA1c levels, reduced depressive symptoms and relieved physical and emotional symptoms. For the current proposal, we enlisted the use of the RE-AIM (reach, effectiveness, adoption, implementation, maintenance) framework, a hybrid evaluation model of the real-world impact of health behavior interventions that provides an evaluative framework for measuring effectiveness of complex, behavioral interventions. Using this model will allow us to assess the effectiveness of the HOPE intervention, while also allowing us to address potential barriers to implementation.

Specific aims are:

1.) To compare at 6 and 12 months the clinical effectiveness of HOPE enhanced primary care with that of education enhanced primary care (EUC).

Hypothesis 1a: After 6 months' active treatment, HOPE will produce greater improvements than EUC in diabetes (shown by glycated hemoglobin [HbA1c] levels) and depression (shown by PHQ-9 scores).

Hypothesis 1b: At 12 months (following a 6-month maintenance phase), HOPE participants will continue to show significantly greater improvements than EUC patients in HbA1c and depression symptoms.

Exploratory Aims

2.) To examine the role of potential moderators and mediators on intervention effectiveness. Exploratory Aim 2a. To evaluate factors that mediate or moderate effectiveness at 6 and 12 months for all enrolled patients. Potential mediating and moderating variables include patient-level (diabetes distress and self-efficacy and sociodemographics) and facility-level (availability of mental health services) factors. Exploratory Aim 2b. To evaluate factors that mediate or moderate effectiveness at 6 and 12 months for patients in the HOPE arm. Factors include adherence (e.g., session attendance), fidelity (ratings of coach effectiveness), and treatment implementation (e.g., goal-setting quality and self-management behaviors).

3.) Evaluate the potential for embedding HOPE in VA primary care settings, using the RE-AIM framework for evaluating effectiveness of behavioral interventions. Exploratory Aim 3a - Reach. Compare clinical and demographic characteristics of participants with those of all potentially eligible patients. Exploratory Aim 3b - Adoption. Qualitatively elicit PACT clinician and participant perceptions of HOPE to prepare for implementation next steps.

Section E: Protocol Risks/Subjects

E1. Risk Category

Category 1: Research not involving greater than minimum risk.

E2. Subjects

Gender:
Both

Age:
Adult (18-64 yrs), Geriatric (65+ yrs)

Ethnicity:
All Ethnicities

Primary Language:
English

Groups to be recruited will include:
Healthy, non-patient, normals; Patients

Which if any of the following vulnerable populations will be recruited as subjects?
Employees or lab personnel

Vulnerable populations require special protections. How will you obtain informed consent, protect subject confidentiality, and prevent undue coercion?

To guard against any undue influence or coercion by the study on the administrating institution's employees, the consent process will emphasize the voluntary nature of the research by including the following statements in the consent form: Participation in this study is voluntary and will not affect your current or future employment status. There is no penalty for refusing to participate and you may withdraw your participation in the study at any time. Additionally, your identifying information and any opinions, insights or information you share will be kept strictly confidential.

E3. Pregnant woman/fetus

Will pregnant women and/or fetuses (as described in 45 CFR 46 Subpart B) be enrolled in the research?
No

E4. Neonates

Will neonates of uncertain viability or nonviable neonates (as described in 45 CFR 46 Subpart B) be enrolled in the research?
No

E5. Children

Will children be enrolled in the research?
No

Section F: Design/Procedure

F1. Design

Select one category that most adequately describes your research:
z.r) Randomized, Efficacy Study -- Surgical Techniques/Interventions

Discuss the research design including but not limited to such issues as: probability of group assignment, potential for subject to be randomized to placebo group, use of control subjects, etc.

We propose a randomized clinical trial to compare the effectiveness of HOPE with that of enhanced usual primary care (EUC) for distance-based Veterans with comorbid diabetes and depressive symptoms receiving

primary care services at the VA. Randomization will occur at the patient level and be stratified by clinical site. In addition, we will evaluate the durability of the treatment over a 6-month period following the active-treatment phase. To better understand the mechanics of change, we will conduct an exploratory examination of the mediating and moderating variables associated with changes in the primary outcomes (HbA1c levels, depression symptoms) at both 6 and 12 months. Additional exploratory analyses will attempt to better understand the relative strengths, weakness, barriers, and facilitators important to future implementation if the intervention produces significant changes.

The proposed study will enroll 242 patients with diabetes and depressive symptoms who receive care in VA CBOCs throughout Southeast Texas, as well as MEDVAMC patients living >20 miles from the hospital who face similar distance related treatment barriers. 60% of the participants (n=146) will be randomized to receive 9 behavioral coaching sessions in 6 months (active intervention), followed by 6 months without coaching (maintenance period); the other 40% (n=96) will receive EUC, which will include the provision of educational materials and feedback about diabetes and depression. All consented participants (both treatment arms) and their PCPs will be notified of the diabetes control and depressive symptoms and provided with guideline-based recommendations for treatment of depressive symptoms. Randomization will be stratified by clinical site.

Inclusion Criteria:

Patient inclusion criteria consist of: (a) a diagnosis of diabetes mellitus; (b) an average HbA1c level >7.5% in the prior 12 months; and (c) clinically significant symptoms of depression. Verification of diabetes mellitus diagnoses will be based on data collected from the VA data warehouse. To verify that participants meet the depression criteria, we will use participant self-report of clinically significant depressive symptoms according to the PHQ-9, where a score of greater than/equal to 10 on the PHQ-9 will signify a clinically meaningful symptom burden.

Coach inclusion criteria consist of: (a) being a VA primary care clinician; and (b) being willing and able to commit to at least 7 months of participation in the study, including training and seeing patients.

Exclusion Criteria:

We will exclude potential participants only for clinical factors that would render a telephone-based behavioral activation intervention inappropriate. Specific exclusion criteria are: (a) lack of regular access to a telephone; (b) significant cognitive impairment (three or more errors) on an established six-item screening exam; and (c) meeting criteria for bipolar, psychotic, or substance-abuse disorders; (d) presence of hearing impairment; (e) their medical chart recommends not titrating therapy due to prior history of significant hypoglycemic events; or (f) they live within 20 miles of the MEDVAMC. Patients will be secondarily excluded if their HbA1C level falls below 7.5% at baseline assessment, or if they report suicidal ideation on the PHQ-9 at baseline assessment.

Patients receiving mental health services for conditions other than bipolar, psychotic or substance-abuse disorders at the time of study recruitment will not be excluded. All mental health treatments and health service-use characteristics will be included in study analyses as covariates.

Coaches do not have any exclusion criteria. All disciplines in primary care are invited to participate.

F2. Procedure

Consenting Clinicians: Before training, interested clinicians will sign the 10-1086 form with study RAs. Consent is necessary as we intend to publish this data. Coaches will act within their regular duties and will not provide any PHI, but will contribute data about the process/experience and implementation of the research. Training and Evaluating Coaches: Coaches may/may not be mental health clinicians, but have a basic background in behavioral sciences. Training will use a 2-step process, involving dedicated workshop-style sessions and ongoing consultation+feedback. Specific procedures include: clinician manual-guided instruction, modeling and role-playing, and regular feedback about ongoing cases. Workshop training sessions will be standardized by the study PIs and occur prior to coaching. Feedback will be structured around the ACE scales developed for the project (Sect S). Oral+written feedback will be offered (form attached in Sect S). Training materials and procedural support will also be made available on an ongoing basis through study website- http://www.vaprojecthope.org/. Access to For Coaches section will be password protected. Data collection from Coaches: Coaches will complete 4 surveys (Sect S) about the process/implementation of the intervention in their clinical setting. Coaches will be assigned an ID#, and the link between ID and names will be stored on a password-protected M drive folder accessible only to study staff not involved in data analysis. Data will be collected using IDs at 4 times: before training workshop, after

training workshop, after feedback with study staff (~6mos post-workshop), at exit from the study. Coaches also complete 2 surveys about experience with each veteran (Sect. S, WAI-C and CEC-C) to be administered~4 mos. after assignment. Survey responses will be entered into a fillable form on a password protected M: drive folder accessible to study staff and coaches (a separate folder from that which contains ID-name link). No PHI or sensitive data will be entered. Once complete, surveys will be entered by staff into a database on the M drive for analysis. Identifying Patient Participants: The study sample will be identified and enrolled using a multi-step screening process. An initial pool of eligible patients with diabetes will first be identified by VISN 16 Data Warehouse and randomly assigned as potential participant lists stratified by clinical site. Information/PHI to be used in this initial data pull that may identify a subject include name, address, phone #, year of diabetes dx and the dates/values of most recent A1c. This info will be used to determine poor glycemic control as an inclusion criterion and to contact the subject to complete the next steps in the screening process. Birth date, race, ethnicity, and gender will also be collected in the initial data pull as descriptive statistics to determine if there are statistically significant differences between interested and not interested patients. The location of the patient’s PC facility and their doctor's name will be collected in the initial data pull to identify subjects to guide randomization and coach pairing after randomization respectively. A waiver of informed consent/HIPAA authorization has been approved by the IRB on 2/15/2012 for this initial step of the screening process (Sect S). We will take all measures necessary to ensure the confidentiality/anonymity of all participants. Patients who meet criteria for uncontrolled diabetes and geographic location will receive an opt-out letter about the study. Patients who do not opt out will be contacted by study personnel for the second step of the multi-step screening process. Study RAs will then use a structured, phone-based screening interview to identify patients who preliminarily meet our depression inclusion criteria on the abbreviated PHQ-8 scale. The screening interview will also exclude from that group who screens positive on the PHQ-8 but for whom a phone-based intervention would be inappropriate. Specific procedures and measures to be administered can be found in HOPEIIR Screening Procedures (Sect S). After completing the detailed screening, patients who still meet eligibility will be approached for informed consent and a baseline assessment. The PHQ-9, as part of the baseline assessment, will serve as the final eligibility check in this multi-step screening procedure. Following the phone screening, interested and preliminarily eligible patients will be mailed a copy of the ICF w/out signature lines, along with the baseline measures. After the patient has time to review these docs, study staff will call the patient on the phone to go over the informed consent process. The patient will have the opportunity to ask questions and must demonstrate an understanding of the risks and benefits of participation before verbal consent to participate is achieved. After consent, the subject will begin the baseline assessment. Because this intervention will be used in the medical care of participants, subject's participation in this protocol will result in their medical records being flagged. The following Title 38 U.S.C. 7332 protected information will be collected for scientific research purposes as part of this research protocol: drug and/or alcohol abuse. This info will be maintained in accordance with the security requirements of 38 CFR Section 1.466, or more stringent requirements. This info will not be re-disclosed except back to the VA. This info will not identify any individual patient or research subject in any report of the research, or otherwise disclose patient or research subject identities. The purpose of collecting alcohol and or drug abuse and treatment info (38USC7332) is to conduct scientific research. No personnel involved, in this study, will identify, directly or indirectly, any individual patient or subject in any report of such research or otherwise disclose patient or subject identities in any manner. Baseline Assessment: Subjects who meet eligibility criteria during the phone screening procedures and give verbal consent will participate in a baseline assessment. All assessments (baseline/ 6- /12-month f/u) will be completed by a blinded independent evaluator (IE) over the phone. Baselines will begin with a socio-demographic survey, followed by the PHQ-9 and a myriad of other measures (see HOPEIIR Baseline and Follow Up Measures document in Sect S). A1c collection will be coordinated by IEs. Lab collection is feasible, with high completion rates in our prior VA studies. In EPIC, A1c collection was higher than survey collection rates. All participants who do not meet the final A1c eligibility criteria but were identified as having significant depressive symptoms will be notified of their results, and a note will be placed in their medical record indicating this finding to their PCP. Enrolled participants will also have a note placed in their medical record indicating the presence of clinically significant depressive symptoms and the results of their most recent A1c labs. The participant will be provided with materials on diabetes and depression, and the participant and PCP will be given evidence-based recs for treatment of the depressive symptoms. After baseline, participants will receive $30. Measures will be repeated from the baseline assessment at 6 and 12-mos., but no demographics. If staff cannot reach a veteran 3x by phone to schedule f/up, a letter may be sent. Packets will be mailed to participants as a courtesy for each assessment so that they may follow along with the measures as they are administered by phone (Sect S). Randomization: All consented participants who complete the baseline and are eligible after baseline and blood draw will be randomized to HOPE or Enhanced Usual Care. Eligible participants at each site will be randomly assigned by a RA following the baseline evaluation. In accordance with VA policy and procedures, participants randomized to the HOPE intervention will receive VHA Form 10-3203 (Sect S) with

their intervention materials in the mail. Participants randomized to HOPE will be instructed to complete the consent for voice recording form and return it in a pre-provided postage paid envelope. Randomization will be blocked and stratified by site to assure balance in the number of patients assigned to HOPE or EUC at each facility. Within each site, we will use random blocks of 4 and 6. The random numbers will be generated in SAS PROC PLAN by Dr. Petersen. The number of patients randomized at each site will be proportional to the number of patients estimated to be eligible for the study at that facility and adjusted as new CBOCs open and their data becomes available. Should a proportional recruitment prove difficult, we will reconsider this practice to ensure that we meet our sample size goals. Conducting EUC: All enrolled participants will receive the EUC procedures at their clinical site pertaining to diabetes care and depressive symptom management. Once participants are consented and enrolled, study personnel will: 1-Send a standardized letter to study participants notifying them of the study findings related to their diabetes A1C levels and depressive symptoms, and a reminder that their PCPs will be notified as per the informed consent (in Sect S as EUC Cover Letter). Patients will also be provided with basic educ. materials related to diabetes and depression (Sect S); 2-Enter a parallel note in the patient’s CPRS record notifying the PCP of these findings, with an add'l signer request (to signify awareness of note); 3-Include recs for MH referral or treatment prompt for depression in the CPRS note; Repeat steps 1-3 after the results of the 6mo and 12mo data collection are available. No specific recs for diabetes care will be provided, no further contact will be made during the acute intervention period for participants in EUC. Conducting HOPE Intervention: Study RAs will contact participants randomized to HOPE to arrange the first behavioral call w/in a 2-3 week window. The coach will initiate calls at the participant’s time and day of choosing. If a veteran fails to appear 3x for the phone appt, a letter may be sent by the staff on behalf of the coach. Like EUC, HOPE patients will receive basic educ info on diabetes and depression. A standardized behavioral counseling framework is used by coaches to guide each of the calls, regardless of the specific contents of each session. At the conclusion of each behavioral coaching call, the coach will summarize the progress of the current session and schedule the next call. The coach will then enter a note in the participant’s CPRS record with the participant’s PCP listed as an additional signer. This note will summarize the specifics of what was discussed during the prior coaching call and provide, in the participant’s own language, the agreed upon specific goal and action plan(s). Coaches may also suggest additional f/u or referrals to the PCP, based on the participant’s needs and requests, such as a referral to a diabetes educator or nutritionist. Coaches may also ask PCPs to schedule a quicker (urgent care) f/u visit if warranted. The actual request for referrals and all medical f/u appts and medication changes will be left to PCPs’ discretion. HOPE Intervention modules: A total of 9 sessions will be conducted during a 6mo period. For the first 3mo, coaching sessions will be 30min, take place bi-weekly, and will include 2 core sessions and 4 elective sessions (where patient chooses topic to cover). In the next 3mo, 3 f/u sessions, each 15min long, will occur once a month. There are a total of 6 modules for the patient to choose from for their elective sessions, and modules may last more than one session. The modules assigned to elective sessions 3-6 are adaptable, and one or more may be presented in any order at the participant’s and coach’s discretion.

Section G: Sample Size/Data Analysis

G1. Sample Size

How many subjects (or specimens, or charts) will be used in this study?

Local: 455 Worldwide: 455

Please indicate why you chose the sample size proposed:

The proposed sample size of 455 includes 425 patients and 30 clinicians.

The sample size of clinicians was determined by creating a schedule for the expected flow of Veteran participants in the study that predicts expected demand for coaching.

In determining the sample size of patients for the study, we examined the number of patients needed to have adequate power for both hypotheses related to the primary aim (specific aim 1). The sample size determined for the primary aim was inflated to account for potential correlation of patients treated by the same PCP and to account for attrition at 1 year of 25%, based on our and other prior VA studies.

The sample size is based on testing for a clinically important difference between the two groups at a single time point, using a two-group t test with a 0.05 two-sided significance level and power of 80%. For HbA1C, a difference of 0.5 is considered clinically significant within a 6-month time frame. Using a difference score of 0.5 in the mean HbA1c between the two groups and an estimated pooled standard deviation of 1.1 results in

an effect size, d = .45. This effect size is consistent with our prior RCT study on behavioral coaching interventions which generated HbA1c level differences of 0.68 and a medium effect size of 0.50 and estimates from our HOPE pilot confirm these effects. Using these data, a sample size of 79 per group or 158 total would be needed for a significant difference in the proportion of participants achieving greater than or equal to 0.5 improvement in HbA1c.

For PHQ-9, a score of less than 10 or a 5-point decline from baseline would be considered a clinically significant improvement. The sample size of 79 per group would have 80% power to detect a change as small as 1.5 points in the PHQ-9 score with a two-sided alpha=0.05. From our own work using cognitive-behavioral therapy for medically ill Veterans with depression, we found clinically significant improvements in Beck Depression Scale scores consistent with effect sizes of 0.98 at 8 weeks and 1.05 at 20 weeks post-intervention. This level of efficacy is consistent with our estimates of clinically significant change as indicated by PHQ-9 scores.

Following the approach of Schnurr et al, we inflated the sample size to account for a correlation among patients seen by the same PCP using the formula, $f = 1 + (m - 1)ñ$, where m is the average number of patients per provider and ñ is the intra-class correlation coefficient. Assuming there will be 40 to 50 PCPs results in m = 4 and estimating ñ as .05 yields f = 1.15. This inflation factor is modest but reasonable, given the nature of the study and the extent of the external clinical providers' involvement (supported by personal communication with Paula Schnurr – May 2010).

Thus, the sample size needed for analysis is 182 total (91 per group). Finally, adjusting this for 25% attrition using the inflation factor of 1.33 [i.e., 1/(1-.25)] gives a total of 242 patients to be recruited. Therefore, a final cohort of 182 at 12-month follow-up (i.e., recruitment of 242 to allow for 25% attrition) would provide sufficient power to conduct analyses for Aim 1. This sample would provide 80% power to detect an effect size of 0.45, which represents a difference in HbA1c of 0.5 with a standard deviation of 1.1 and a difference in PHQ-9 scores of as little as 1.5 with standard deviation of 3.4.

G2. Data Analysis

Provide a description of your plan for data analysis. State the types of comparisons you plan (e.g. comparison of means, comparison of proportions, regressions, analysis of variance). Which is the PRIMARY comparison/analysis? How will the analyses proposed relate to the primary purposes of your study?

Data Collection Strategy: Data will be collected by self-report, chart review, and laboratory collection. Independent Evaluators (IE) who are blinded to the study procedures and treatment assignments will collect data at 6 and 12 months by phone. They will mail assessment packets to participants and then work with them to complete all self-report items. IEs will arrange a lab visit for a blood draw within 2 weeks before and 2 weeks after the target data-collection time (facilitated by PCPs). If PCPs have already obtained A1c levels in that timeframe, those values will be used. Participants will receive modest compensation after A1c results and self-report assessments are collected. RAs will also perform chart abstraction of clinical baseline, moderating, and exploratory variables. Data Quality Assurance: Using previously developed and standardized procedure manuals, providing rigorous training and fidelity measurement of study coaches and independent evaluators, as well as providing written guides for assessments are ways quality will be assured. Attrition/Retention and Missing Data: We expect rates of missing data for primary outcomes less than 25%, leading to primary outcome data for greater than 75% of participants. To evaluate the potential relationship between loss to f/u and outcome variables, reasons for study discontinuation will be recorded for subsequent analyses, and overall attrition rates will be factored in the analyses of the primary specific aim. We will conduct sensitivity analyses using tests for data missing completely at random and tests for nonrandom missingness. We will also plot the data over time to visually assess changes in outcomes from baseline to 12 mos. and to indicate whether additional terms are needed in the models to account for nonlinearity over time. Data Analysis Plan: Primary Analyses: A1c and Depression Outcomes--We will compare participants in the two study arms on baseline characteristics, including sociodemographic characteristics (age, race/ethnicity, gender, education level, independent living status, VA copay status, employment status) and clinical variables (BMI, Deyo comorbidity score, psychotropic and diabetes medications and treatments, specific psychiatric diagnoses, and number of PCP visits in the previous 12 mos). Based on recommendations of the CONSORT group, we will not provide tests of significance or adjust subsequent analyses for variables found to differ because adjustment may bias the estimated treatment effect. However, the analyses for the primary aim will be adjusted for variables we have decided a priori may be important to control: a) baseline values of HbA1c and PHQ-9, b) whether the patient was on exogenous insulin, and c) the stratification variable, site. In addition, we will compare participants on d) access to psychotherapy and medication, defined as dichotomous variables. At baseline, 6 months, and 12 months, we will construct variables for whether the patient was receiving

psychotherapy during the previous 6 months (yes/no), whether psychotropic medications were started or changed during the past 6 months (yes/no), and whether insulin was added during the past 6 months (yes/no). A1c and Depression Outcomes at 6 months--The patients' A1c levels and PHQ-9 scores at 6 months will be the primary outcomes. We will examine the normality of the distributions of these outcomes and will consider transformations such as the log or the inverse, if necessary. Separate models will be run for HbA1c and for PHQ-9. We will run hierarchical linear model analyses to account for the correlation of patients being seen by the same PCP. Using this nested approach, we will test if there are differences between EUC and the HOPE intervention, using the A1c level and the PHQ-9 score (either untransformed or transformed, if needed) as dependent measures. Although patients are also nested within coach, the coaches are not assigned exclusively to certain PCPs. We will include intervention fidelity scores for each coach as a study variable in our analysis of this aim to account for coaching fidelity effects. Each model will contain a variable indicating the treatment group, indicator variables representing site, whether or not the patient was on exogenous insulin (evidence suggests that insulin use impacts both A1c and depressive symptoms), receiving psychotherapy, taking psychotropic meds, and baseline A1c or PHQ-9 levels as independent variables. A significant value for the treatment group variable in the hierarchical model will indicate that there were statistically significant differences between the intervention and EUC. In addition, we will examine whether the groups differed as to whether or not they had clinically significant changes in A1c and PHQ-9 by dichotomizing the outcomes. A significant decrease for A1c is defined as 0.5 between each time point and from baseline to 12-months. For PHQ-9, this will be a change score of 5 or greater at 6- and 12-months. We will test whether the two groups (clinically significant change vs. no change) differed using a hierarchical logistic-regression model in which the dichotomous outcomes are the dependent variable. The independent variables will be those described above for the hierarchical linear regression. A1c and Depression Outcomes at 12-months-- The patients' A1c levels and PHQ-9 scores at 12-months will be the primary outcomes. Similar to the analyses at 6 months, we will examine the distribution and run hierarchical linear models on the 12 month data. In addition, we will examine all of the time periods in a hierarchical longitudinal analysis, separately for A1c and PHQ-9. This random coefficient model will allow us to fit a line for each participant using his/her available data, including participants with missing values, and will maximize the power to detect differences because patients are not removed from the analysis if any of the time periods is missing. Patients will be nested with their PCP. The model will contain the A1c and PHQ-9 values for each of the time periods (baseline, 6 and 12 months). The independent variables in the model will be time, treatment, the interaction of time and treatment, site, and use of exogenous insulin psychotropic meds, or psychotherapy. The treatment effect will measure differences between the intervention and EUC groups at baseline, and the time effect will measure whether there was an overall change over time in the two clinical outcomes. The time-by-treatment interaction is of most interest in the longitudinal analysis because a significant value will indicate that there was a difference between the intervention and EUC groups over the 12-month time period. Mediators and Moderators: To examine the role of potential moderators and mediators on intervention effectiveness, we will evaluate factors that mediate and moderate outcomes at 6 and 12 months. Moderating variables are those that specify on whom and under which conditions treatments have different effects. The moderating variables for this aim include sociodemographic traits, BMI, medical and psychiatric comorbidity, and social support. Mediator variables identify possible mechanisms through which a treatment achieves its effect. Mediators include the GET-D scores for goals and action plans, diabetes-related distress, self-efficacy, performance of self-care behaviors, and adherence to pharmacological therapy. We will consider the difference between the moderators and mediators in terms of when they occur relative to treatment and whether they are correlated with treatment. Moderators must precede treatment and should not be correlated with treatment. Mediators will occur during treatment and should be correlated with treatment. Thus, we will initially test for the association of the moderating and mediating variables with treatment. Since the treatment is a dichotomous variable, we will use t tests to determine if continuous moderators or mediators are associated with treatment and chi-square tests to determine if ordinal or categorical moderators or mediators are related to treatment. After verifying that a possible moderator is not significantly related to treatment, we will run the model, including a term in the model for the moderator and a term for the interaction of that variable with treatment. A significant value for the interaction term will indicate that the effect of treatment on the individual patients depends on their value of the moderator. For mediators, we will test if the variable has either a main or interactive effect on the outcome. With an emphasis on the effect size of treatments and what influences the effect size for a particular moderator or mediator, we will calculate effect sizes at each of the scheduled time points. In addition, we will form response categories to assess the stability of variables under two approaches to measuring outcomes (one using continuous variables for outcomes and the second using categories for the outcomes). For example, we might define an improvement in A1c as a decrease of 0.5 from baseline or PHQ-9 decrease of 5 from baseline. We would then run the analytic models using the dichotomous variable of whether or not the patients' A1c level improved as the outcome variable. If the two-variable presentations prove to be similar, we will likely use the categorical approach because of greater ease in reporting results to

clinicians based on response categories. Evaluating the potential for embedding the HOPE intervention within VA (Assessing Implementation): Exploration of reach--We will compare the demographic and clinical characteristics of study participants from the study sites to those of potentially eligible patients typically cared for at each CBOC and the MEDVAMC. For each site, we will test whether patients who participated differed from (a) patients who were approached but refused enrollment and (b) all remaining patients who would have met inclusion criteria but who were not included in the study, using analysis of variance for continuous variables and chi-square tests for categorical variables. The variables of interest will include age, gender, race, BMI, Deyo comorbidity score, and presence of PTSD or major depression. These variables will be obtained from the VISN 16 data warehouse. The potentially eligible patients cared for at each facility will be determined using diagnoses of diabetes and depression from the inpatient and outpatient files and A1c values from the VISN 16 data warehouse (with IRB approval). Exploration of adoption--We will evaluate the perceptions and practices of clinicians regarding intervention processes including the incorporation of behavioral goals and action plans within the progress notes and treatment plans described by PCPs. To accomplish this aim, we will purposively sample PCPs at each clinical site, including those who have cared for control and intervention subjects. We will use respondents' PACIC scores to purposively sample low and highly rated PCPs on the patient engagement and goal-setting subscales. Recruited PCPs will be evaluated using in-depth interviews to elicit their perceptions of the overall intervention procedures and their use of any constituent elements of each intervention. Consistent with established qualitative interview methodology, interviews will be audiotaped, transcribed, and analyzed using a deductive approach based on a coding system developed from our interview guide. Coded units will be reexamined for relationships and clustered into patterns. Based on past experience, we anticipate that each interview will last 30 minutes. Quantitative analyses will also be done on the coach surveys completed pre-, post-, and 6-months-post-training to assess change over time. The results of these quantitative and qualitative analyses will guide future implementation studies of the HOPE intervention in usual VA care.

Section H: Potential Risks/Discomforts

H1. Potential Risks/Discomforts

Describe and assess any potential risks/discomforts; (physical, psychological, social, legal, or other) and assess the likelihood and seriousness of such risks:

For patients, this trial poses minimal risk; however, there are still some potential risks associated with the proposed tests to assess the impact of the intervention, as well as the intervention itself. Risks associated with the assessments are low given that the items assessed are normal daily activities including blood draws that are conducted as part of the standard of care. Our eligibility criteria will exclude patients with mental health disorders (e.g. actively suicidal, biopolar, psychotic, etc.). We will screen subjects directly using a 6-item screening exam. Thus, using this multi-gated approach, we should be able to effectively screen-out any individuals for whom this intervention is contraindicated. Workbooks also will include a listing of warning signs with instructions should injury or other health problems occur. Study participants will be provided a telephone number in order to communicate with study staff. All health occurrences will be recorded and regularly reviewed by the study staff as well as the VA and BCM Institutional Review Boards, as applicable. For clinicians, the possibility of any potential risks or discomforts is considered minimal for this project because coaching is part of their regular clinical duties. There is a small possibility for loss of confidentiality, although coach participants will be discouraged from addressing themselves by name during the exit interview or surveys to mitigate that risk. Additionally, coaches will be assigned unique, study ID#s, and the independent evaluators and research staff doing analyses will be blinded to coach identity.

H2. Data and safety monitoring plan

Do the study activities impart greater than minimal risk to subjects?

No

H3. Coordination of information among sites for multi-site research

Is the BCM Principal Investigator acting as the SPONSOR-INVESTIGATOR for this multi-site research?

No or Not Applicable

Is BCM the COORDINATING CENTER for this multi-site research?

No or Not Applicable

Section I: Potential Benefits

Describe potential benefit(s) to be gained by the individual subject as a result of participating in the planned work.

Patients may develop skills to set high-quality treatment goals and action plans targeting diabetes and depression self-care. This intervention will potentially improve participants' overall health, and behaviors for diabetes and depression. Patients will receive appropriate treatment based on their lab results and the results of the behavioral health assessment will be provided to the patients' primary healthcare provider.

Coach participants will be trained in providing effective behavioral health coaching, which will contribute to their professional development and may provide benefit for their clinical practice outside of this intervention.

Describe potential benefit(s) to society of the planned work.

The establishment of a feasible and valid behavioral health coaching intervention for patients with diabetes and depression.

Do anticipated benefits outweigh potential risks? Discuss the risk-to-benefit ratio.

The risks are minimal and are greatly outweighed by the benefits. The risk-to-benefit ratio is favorable.

Section J: Consent Procedures

J1. Waiver of Consent

Will any portion of this research require a waiver of consent and authorization?

Yes

Please describe the portion of the research for which a waiver is required. (Example: chart review to determine subject eligibility)

A waiver of consent and authorization is required in order to conduct chart reviews to identify potentially eligible participants for the study. Once identified, subjects will be mailed an opt out letter. * Please note that the study is no longer recruiting participants *

Explain why the research and the use or disclosure of protected health information involves no more than minimal risk (including privacy risks) to the individuals.

The waiver is being requested to only identify potentially eligible Veterans. Veterans will be mailed an opt-out letter and be given the opportunity to call in to decline participation. Veterans that don't opt out, will be contacted for a screening call and will be explained that participation is strictly voluntary. After confirmation at the screening call, Veterans will be scheduled for a baseline assessment. At the assessment a complete description of the study will be provided and Veterans will have the option to decline before study related data is collected. Any data collected before the baseline assessment will not be used from Veterans that declined participation.

Explain why the waiver will not adversely affect the privacy rights and the welfare of the research subjects.

A waiver of the informed consent requirement for recruitment purposes will not adversely affect the rights or welfare of Veteran participants. The waiver is only being requested to conduct chart reviews to exclude Veterans not eligible for the study. Veterans that are not excluded will be mailed an opt out letter. At the time of the screening call Veteran's will be explained the study and that participation is completely voluntary and their decision to participate or not will not affect the service-related right to which they may be entitled. Additionally, at the scheduled baseline study staff will review the detailed information document providing another opportunity for Veteran participants to decline participation.

Explain why the research could not practicably be conducted without the waiver and could not practicably be conducted without access to and use of the protected health information.

The project has a specific focus on patients with a diagnosis of diabetes. Therefore, to reduce the undue burden on Veterans that don't have this condition we are requesting the waiver. An initial data extraction from national VA databases will identify the pool of potential study participants who may have the condition by looking for specific ICD- 9 codes for diabetes and that receives care at the Houston hospital and surrounding Community Based Outpatient Clinics (CBOCs).

Once identified, a standardized chart review will identify ineligible patients. Research assistants will access a secure database containing the names and social security numbers of patients identified during the initial data extraction. They will then access these patients' records in CPRS and manually search the active and inactive problems lists for exclusionary diagnoses, including 1) cognitive impairment, 2) bipolar disorder, 3)psychotic disorders, and 4) substance abuse disorders.

Veterans identified to be eligible via the chart review will be contacted for a telephone screening if they 1) do not opt out of contact with study personnel and 2) express interest in participating. During the initial phone contact, a research assistant will administer the screening questions. The RA will follow a script for the telephone contact that includes assent procedures for the screening questions Participants will be asked questions to screen for depression (PHQ-8), cognitive functioning (6 –item Cognitive Screen), presence of bipolar and psychotic disorders, substance use (MINI), and alcohol use (AUDIT-C). Eligible participants (those who screen positive for depression on the PHQ-8 and are not excluded for more severe mental health issues) will progress to the final recruitment procedures. Veterans that are not eligible will not receive any further contact and information collected on them will not be utilized.

Describe how an adequate plan exists in order to protect identifiers from improper use and disclosure.

Each participant will be assigned a unique study ID number that will contain no personally identifiable information. The PI and the Project Coordinator will maintain a master link list of the unique study ID number, the patient name, and the patient SSN in a password-protected file on the Houston VA secure computer server. Study personnel with access to the unique study ID include the study PI (Naik), Project Coordinator, the Main Site (Houston) RA, local site investigator, local site RA, study biostatistician, and study statistical programmer. Any documents that contain personally identifiable information will be kept separate and apart from data collection documents that are coded with the subject ID number.

Describe how an adequate plan exists in order to destroy identifiers at the earliest opportunity consistent with conduct of the research, unless there is a health or research justification for retaining the identifiers or such retention is otherwise required by law.

All research data collected, including identifiers, for this VA research study will be maintained for six years from the date the research study is closed.

Describe how adequate written assurances exist in order to ensure that the PHI will not be reused or disclosed to (shared with) any other person or entity, except as required by law, for authorized oversight of the research study, or for other research for which the use or disclosure of the PHI would be permitted under the Privacy Rule.

Only authorized members of the research team will have access to participant data and information. Data with Protected Health Information (PHI) will be kept on the VA Informatics and Computing Infrastructure (VINCI) server or at the server housed at the Houston VA HSR&D Center of Innovations in Quality, Effectiveness and Safety (IQuEST), where strict privacy rules are implemented. All paper data will be stored in a locked file cabinet located in a secure data storage room that is only accessible via electronic keypad entry. Individual files containing PHI will be password protected and accessible only to authorized study team members. Customized permissions will govern which elements of the data management system that study personnel may access.

Information from health records such as diagnoses, progress notes, medications, lab or radiology findings, etc.

Yes

Specific information concerning alcohol abuse:

No

Specific information concerning drug abuse:

No

Specific information concerning sickle cell anemia:

No

Specific information concerning HIV:

No

Specific information concerning psychiatry notes:

Yes

Demographic information (name, D.O.B., age, gender, race, etc.):
Yes

Full Social Security #:
Yes

Partial Social Security # (Last four digits):
Yes

Billing or financial records:
No

Photographs, videotapes, and/or audiotapes of you:
Yes

Other:
No

Will additional pertinent information be provided to subjects after participation?
Yes

If Yes, explain how subjects will be provided additional pertinent information after participation.
Patient subjects will be provided with feedback on the information collected during the study. They will also be given the results of their lab work as the study progresses. Study subjects will receive personalized information regarding their diabetes and depression management, according to the intervention modules.

J1a. Waiver of requirement for written documentation of Consent

Will this research require a waiver of the requirement for written documentation of informed consent?
Yes

Explain how the research involves no more than minimal risk to the participants, and the specifics demonstrating that the research does not involve procedures for which written consent is normally required outside of the research context.

We are requesting permission to verbally consent patients to this study by telephone. Given the rural focus and exclusively telephone-based study procedures, a verbal consent process will afford us greater opportunities to increase our outreach to these at risk patients, while also reducing the burden on patients. We received approval to conduct a screening appointment via a waiver of requirement for written documentation of consent for our prior pilot study (#H-26812) using the same protocol proposed for this study. Consistent with this prior study, patients will be initially introduced to the study and screened by telephone. If the initial screen indicates the patient may be eligible for the study, the patient will be mailed detailed information about the study procedures and an informed consent document (without signature lines) for their review. The study staff will then follow up with the patient by telephone and proceed to review all informed consent materials before administering the final battery of inclusion and baseline measures. The study staff will record the date of verbal consent in the research enrollment log. We are also requesting to waive HIPAA authorization from subjects because we will be conducting the consent process and intervention over the telephone. As the pilot study was conducted before the release of the new VA Handbook 1200.05, there was no previous procedural need to record a HIPAA authorization separate from the consent. The grant supporting this protocol was funded and financially organized under the premise that all enrollment procedures would be conducted over the telephone. As such, the window for recruitment is set at 2 years. Given the change in the VA¿s procedures, the conditional requirement of a physical signature on the HIPAA authorization before commencing the baseline assessment and establishing final eligibility represents a significant challenge to the minimum recruitment expectations of this study. The deficits in self-management imposed on these subjects by the concurrent symptoms of their depression and diabetes present significant difficulties that render even interested subjects extremely unlikely to sign and return the form. The resulting delay in enrollment would make it impossible to enroll significant enough numbers of eligible subjects to allow for generalizable analysis of this work within the prescribed 2 year window for recruitment. The external

validity and much of the value in this research is thereby compromised by the requirement for a written HIPAA authorization. Accordingly, we request a waiver of HIPAA authorization for this verbal consent process.

Any personal identifiable data will be stored separate from study data behind two locks in a cabinet within the PI¿s office. Access to these files will be restricted. Our protections for confidentiality are described in detail below. The telephone screening will be conducted using a structured script (attached in section S). Screening will include measures of cognition, depressive symptoms, and psychiatric symptoms. Screening of depression using the PHQ-8 may rarely identify patients having suicidal ideation during this screening process. When this occurs, patients will be referred to one of the study PIs (both credentialed providers at the MEDVAMC). Using a pre-established protocol, Drs. Naik and Cully will contact the patient and make arrangements for immediate mental health assessment and treatment based on the patient's symptoms.

J2. Consent Procedures

Who will recruit subjects for this study?

PI's staff

Describe how research population will be identified, recruitment procedures, any waiting period between informing the prospective participant and obtaining consent, steps taken to minimize the possibility of coercion or undue influence and consent procedures in detail.

Clinicians will self-identify from presentations by study staff in primary care clinics. Study staff will arrange a private meeting with interested clinicians, where they will review and sign the 10-1086 together. This may be done immediately after the presentation or at a later date, depending on clinician availability and preference. The clinicians will have a chance to hear all the details of the study and ask questions, and will be provided with a copy of the signed ICF. We will use the VISN 16 data warehouse to identify potentially eligible patients by these steps: 1. Identify patients with a PCP visit during the past year at the VAMC and/or the CBOCs. 2. Search for patients with type 2 diabetes by ICD-9 codes. A standardized medical record review will confirm diabetes (DM) dx and evidence of exclusion criteria. 3. After verification, patients will be contacted for initial recruitment via a mailed opt-out letter. Letters, written at a 6th grade reading level, will indicate that research personnel will contact the patient after 4 days unless the patient calls the given 800# to opt-out, as directed by the letter. 4. After waiting, a delegated researcher will contact patients to administer a screening protocol. Those who still meet criteria will be mailed a baseline packet and an ICF w/out signature lines. A delegated IE will then call to review the documents and collect verbal consent. Patients who pass the initial screening and consent to participate but are subsequently excluded by the baseline inclusion criterion (PHQ9 or A1c) may be re-contacted at a later date. If interested, the patients will be re-assessed with the PHQ-9 and/or A1c measure to see if they have become eligible for randomization. A waiver of consent will be requested to initiate the medical record review using the VISN 16 data warehouse. Specifications for the waiver are: a) Explain why the use or disclosure of PHI involves no more than minimal risk to the individuals and that the waiver will not adversely affect the privacy rights and the welfare of the individuals. The waiver of consent will only be used to identify a) dx of DM; b) level of DM control; and c) telephone contact info. We will use this info to identify potentially eligible patients and approach them for informed consent. Patients will be sent an opt-out letter prior to any phone contact. For those patients who opt out using the 800#, all PHI will be erased from our files. Patients who do not opt out, will be given the option to provide verbal consent after the waiting period. Those patients who refuse verbal consent will also have their PHI erased from our files. b) Explain why the research could not practicably be conducted w/out the waiver and could not practicably be conducted w/out access to and use of the PHI. Initial access to the PHI is necessary to identify potentially eligible patients. Given our strict eligibility criteria, it would be impossible to recruit our targeted sample of patients w/out first limiting recruitment to uncontrolled diabetics who could then be approached to evaluate for depressive symptoms. These patients rarely enroll in studies w/out first being screened and identified as potential candidates for enrollment. The telephone nature of our screening and intervention procedures requires that we first identify a large sample of potentially eligible patients. Given these circumstances and criteria, our recruitment would be virtually impossible w/out first limiting enrollment to those with known DM with poor A1c control. This can only be done with an initial waiver of consent for the screening criteria. c) Will additional pertinent info be provided to subjects after participation? Yes, Subjects will be provided with feedback on the info collected during the study. They may also request the results of their lab work. Study subjects will receive personalized info regarding their diabetes and depression management, according to the intervention modules.

Are foreign language consent forms required for this protocol?

No

[illegible]

Will the research involve observation or intrusion in situations where the subjects would normally have an expectation of privacy?
Yes

J4. Children

Will children be enrolled in the research?
No

J5. Neonates

Will non-viable neonates or neonates of uncertain viability be involved in research?
No

J6. Consent Capacity - Adults who lack capacity

Will Adult subjects who lack the capacity to give informed consent be enrolled in the research?
No

J7. Prisoners

Will Prisoners be enrolled in the research?
No

Section K: Research Related Health Information and Confidentiality

Will research data include identifiable subject information?
Yes

Information from health records such as diagnoses, progress notes, medications, lab or radiology findings, etc.
Yes

Specific information concerning alcohol abuse:
No

Specific information concerning drug abuse:
No

Specific information concerning sickle cell anemia:
No

Specific information concerning HIV:
No

Specific information concerning psychiatry notes:
Yes

Demographic information (name, D.O.B., age, gender, race, etc.):
Yes

Full Social Security #:
Yes

Partial Social Security # (Last four digits):
Yes

Billing or financial records:
No

Photographs, videotapes, and/or audiotapes of you:
Yes

Other:
No

At what institution will the physical research data be kept?
All data for the study will be housed at the Michael E. DeBakey VA Medical Center.

How will such physical research data be secured?
Each participant (patient and clinician) in the study will receive a unique ID number to maintain anonymity. All paper data will be stored in a locked file cabinet located in a secure data storage room (221) located in the John P. McGovern Campus (Nabisco building), Suite 01Y. Clinician consent forms will be filed in a locked cabinet, separate from all coded study data. Access to research records will be restricted to the PI and his project staff. Audio files of the patients verbal consent and all recorded sessions will be saved with the participant's study ID number. Access to these recordings will also be limited to the study team and stored on VA computers (behind the VA firewall) under Drive:M. All other PHI data will be stored on VA computers (behind the VA firewall) using password protected files.

At what institution will the electronic research data be kept?
Research data for the study will also be housed at the Michael E. DeBakey VA Medical Center. The electronic data is located at: Mdrive\Research\Naik_A_HOPE Depression_H-30139

Research records, including identifiers will be destroyed 6 years after cutoff (at the end of the fiscal year) after completion of the research project, but may be retained longer if required by other federal regulations or sponsor archive requirement.

Such electronic research data will be secured via BCM IT Services- provided secured network storage of electronic research data (Non-Portable devices only):
No

Such electronic research data will be secured via Other:
Yes, (describe below):
The PI and the Project Coordinator will maintain a master link list of the unique study ID number, the patient name, and the patient SSN in a password-protected file on the VA secure computer server. Study personnel with access to the unique study ID include the study PI (Naik), Project Coordinator, RA, biostatistician, and study statistical programmer. Access to drives requires a VA login (username, strong password, and VHA domain). The system administrator restricts the study folders on the Mdrive to be accessed by designated study personnel only.

Will there be anyone besides the PI, the study staff, the IRB and the sponsor, who will have access to identifiable research data?
Yes, identify the classes of the persons:
People who ensure quality from the institutions where the research is being done, federal and other regulatory agencies will have access to all of the research data.

Please describe the methods of transmission of any research data (including PHI, sensitive, and non-sensitive data) to sponsors and/or collaborators.
At no time will data be transmitted outside of the VA. If the study is audited by a federal regulatory agency, the review of the data will occur within our facility using a VA computer.

Will you obtain a Certificate of Confidentiality for this study?
No

Please further discuss any potential confidentiality issues related to this study.
To the best of our knowledge there are no additional potential confidentiality issues related to the study.

Section L: Cost/Payment

Delineate clinical procedures from research procedures. Will subject's insurance (or subject) be responsible for research related costs? If so state for which items subject's insurance (or subject) will be responsible (surgery, device, drugs, etc). If appropriate, discuss the availability of financial counseling.

All medical care provided as part of this study will be authorized by the patient's clinicians and consistent with standards of diabetes and depression care. Study patients' insurance may be billed for clinician visits, lab draws, and related diagnostic tests that are provided as part of standard of care.

If subjects will be paid (money, gift certificates, coupons, etc.) to participate in this research project, please note the total dollar amount (or dollar value amount) and distribution plan (one payment, pro-rated payment, paid upon completion, etc) of the payment.

Dollar Amount:

90

Distribution Plan:

Each patient participant will receive a check for $30 at the completion of each of the 3 assessments: at baseline, 6-months and 12-months. These will be distributed by mail after the completion of the full assessment. Coaches will not receive payment for their participation.

Section M: Genetics

How would you classify your genetic study?

Discuss the potential for psychological, social, and/or physical harm subsequent to participation in this research. Please discuss, considering the following areas: risks to privacy, confidentiality, insurability, employability, immigration status, paternity status, educational opportunities, or social stigma.

Will subjects be offered any type of genetic education or counseling, and if so, who will provide the education or counseling and under what conditions will it be provided? If there is the possibility that a family's pedigree will be presented or published, please describe how you will protect family member's confidentiality?

Section N: Sample Collection

SAMPLE: Blood

What is the purpose of the sample collection?

This study is developing an intervention that is intended to improve diabetes control in patients, measured as HbA1c levels, and depressive symptom outcomes (measured with PHQ-9 questionnaire) using customized behavioral coaching. Blood draws for HbA1c levels will not be done by study personnel and study personnel will not have access to blood samples. Rather, these labs will be done at the patient's VA clinic as part of their standard of care for diabetes. Notably, these blood collections are not being conducted solely for research purposes. Where necessary and requested, the PI (Naik) will work in collaboration with the patient's primary care physician to request lab draws. All such requests will utilize the VA's electronic medical record to communicate with the patient's primary care provider.

For blood draws, specify the amount drawn, in teaspoons, at each visit and across the course of the subjects entire participation time.

Study participants will be encouraged to obtain standard blood draws at baseline and at 6 and 12 months follow-up, as coordinated through their primary care physician and within usual care standards for HbA1c monitoring in diabetic patients. The research personnel will not be drawing labs.

Is there the possibility that cell lines will be developed with this sample?No

Sample will be obtained from:

Other: No labs will be retained by the study team

If sample will be released outside the hospital:

Will sample be released to anyone not listed as an investigator on the protocol? Will the information be identifiable, coded or de-identified?

Will sample material be sold or transferred to any third parties? Will the information be de-identified?

If sample will be banked for future use:

Where will the sample be banked and for how long?

Does the banking institution have an approved policy for the distribution of samples?

If the entire sample will NOT be used during the course of this research study:

Will the remaining tissue be discarded? If not what will be done with the remaining sample after study completion and how long will the sample be kept?

Will samples be made available to the research subject (or his/her medical doctor) for other testing?
No

If a subject withdraws from the study:

Will subject have the option to get the remaining portion of their sample back?
No

Will samples be destroyed? If not, will they be kept anonymously? What will happen to the sample if the subject revokes authorization?

Will data obtained from their sample be deleted? What will happen to the sample if the subject revokes authorization?

Will study data or test results be recorded in the subject's medical records?
No

Will results of specific tests and/or results of the overall study be revealed to the research subject and or his/her doctor?

Please identify all third parties, including the subject's physician, to receive the test results.

Section O: Drug Studies

Does the research involve the use of ANY drug* or biologic? (*A drug is defined as any substance that is used to elicit a pharmacologic or physiologic response whether it is for treatment or diagnostic purposes)
No

Does the research involve the use of ANY gene transfer agent for human gene transfer research?

No

O1. Current Drugs

Is this study placebo-controlled?
No

Will the research involve a radioactive drug that is not approved by the FDA?
No

Section P: Device Studies

Does this research study involve the use of ANY device?
No

Section Q. Consent Form(s)

None

Section R: Advertisements

None
