# Supplementary material for: Effect of Telephone-Delivered Collaborative Goal Setting and Behavioral Activation vs Enhanced Usual Care for Depression Among Adults With Uncontrolled Diabetes: A Randomized Clinical Trial
Source: JAMA Netw Open. 2019 Aug 7;2(8):e198634. doi: 10.1001/jamanetworkopen.2019.8634 (PMC6686779; doi:10.1001/jamanetworkopen.2019.8634)
Supplement: Supplement 2. — Data Sharing Statement [file jamanetwopen-2-e198634-s002.pdf]

Data Sharing Statement

Naik. Effect of Telephone-Delivered Collaborative Goal Setting and Behavioral Activation vs Enhanced Usual Care for Depression Among Adults With Uncontrolled Diabetes. *JAMA Netw Open*. Published August 07, 2019. 10.1001/jamanetworkopen.2019.8634

Data
Data available: No

Additional Information
Explanation for why data not available: VA data privacy limitations
